# Association between serum short-chain fatty acid levels and the risk of all-cause and cardiovascular disease mortality in Chinese patients undergoing maintenance hemodialysis: a retrospective cohort study

**DOI:** 10.1093/ckj/sfaf168

**Published:** 2025-05-29

**Authors:** Xiu-Nan Zhao, Shu-Xin Liu, Shuang Zhang, Zhen-Zhen Wang, Zi Lin, Xue-Lian Jian, Cui Dong, Yi-Nan Zhang

**Affiliations:** Department of Nephrology, Central Hospital of Dalian University of Technology, No. 826, Xinan Road, Dalian, Liaoning, China; Dalian Key Laboratory of Intelligent Blood Purification, Central Hospital of Dalian University of Technology, No. 826, Xinan Road, Dalian, Liaoning, China; Department of Nephrology, Central Hospital of Dalian University of Technology, No. 826, Xinan Road, Dalian, Liaoning, China; Dalian Key Laboratory of Intelligent Blood Purification, Central Hospital of Dalian University of Technology, No. 826, Xinan Road, Dalian, Liaoning, China; School of Clinical Medicine, Faculty of Medicine, Dalian University of Technology, No. 2, Linggong Road, Dalian, Liaoning, China; Department of Nephrology, Central Hospital of Dalian University of Technology, No. 826, Xinan Road, Dalian, Liaoning, China; Dalian Key Laboratory of Intelligent Blood Purification, Central Hospital of Dalian University of Technology, No. 826, Xinan Road, Dalian, Liaoning, China; Department of Nephrology, Central Hospital of Dalian University of Technology, No. 826, Xinan Road, Dalian, Liaoning, China; Dalian Key Laboratory of Intelligent Blood Purification, Central Hospital of Dalian University of Technology, No. 826, Xinan Road, Dalian, Liaoning, China; Department of Nephrology, Central Hospital of Dalian University of Technology, No. 826, Xinan Road, Dalian, Liaoning, China; Dalian Key Laboratory of Intelligent Blood Purification, Central Hospital of Dalian University of Technology, No. 826, Xinan Road, Dalian, Liaoning, China; Department of Nephrology, Central Hospital of Dalian University of Technology, No. 826, Xinan Road, Dalian, Liaoning, China; Dalian Key Laboratory of Intelligent Blood Purification, Central Hospital of Dalian University of Technology, No. 826, Xinan Road, Dalian, Liaoning, China; Department of Nephrology, Central Hospital of Dalian University of Technology, No. 826, Xinan Road, Dalian, Liaoning, China; Dalian Key Laboratory of Intelligent Blood Purification, Central Hospital of Dalian University of Technology, No. 826, Xinan Road, Dalian, Liaoning, China; Department of Nephrology, Central Hospital of Dalian University of Technology, No. 826, Xinan Road, Dalian, Liaoning, China; Dalian Key Laboratory of Intelligent Blood Purification, Central Hospital of Dalian University of Technology, No. 826, Xinan Road, Dalian, Liaoning, China

**Keywords:** butyric acid, cohort study, hemodialysis, mortality, short-chain fatty acids

## Abstract

**Background:**

The association between short-chain fatty acid (SCFA) levels and the risk of all-cause and cardiovascular disease (CVD) mortality in patients undergoing maintenance hemodialysis (MHD) is inconclusive. Furthermore, no studies on the significance of SCFA levels in MHD patients have been conducted in China. Therefore, this association was investigated in MHD patients.

**Methods:**

In this retrospective cohort study, 260 MHD patients were followed up at Central Hospital of Dalian University of Technology between January 2015 and December 2017. Serum SCFA levels were categorized into three tertiles, and the lowest tertile served as the reference group. Survival curves were obtained using the Kaplan–Meier method. Hazard ratios (HRs) and 95% confidence intervals (CIs) were calculated using Cox proportional hazard models.

**Results:**

There were 141 all-cause deaths during the follow-up period of (91.00 ± 0.84) months, of which 85 were due to CVDs. Kaplan–Meier analysis revealed that the risk of CVD mortality in the highest tertile of serum butyric acid level was significantly lower than that in the lowest tertile (log-rank *P *< .05). The level of serum butyric acid was negatively associated with the risk of CVD mortality (HR 0.368, 95% CI 0.187–0.724) after adjusting for potential confounders, and a linear trend was evident in this association (*P* < .05). A linear dose–response relationship was also observed between butyric acid and CVD mortality (*P* nonlinearity >.05). However, none of the SCFAs was associated with the risk of all-cause mortality after adjusting for potential confounders.

**Conclusion:**

Serum butyric acid level was associated with lower risk of CVD mortality among MHD patients. Further prospective large-scale studies are needed to confirm this finding.

## Graphical Abstract

**Graphical Abstract:**
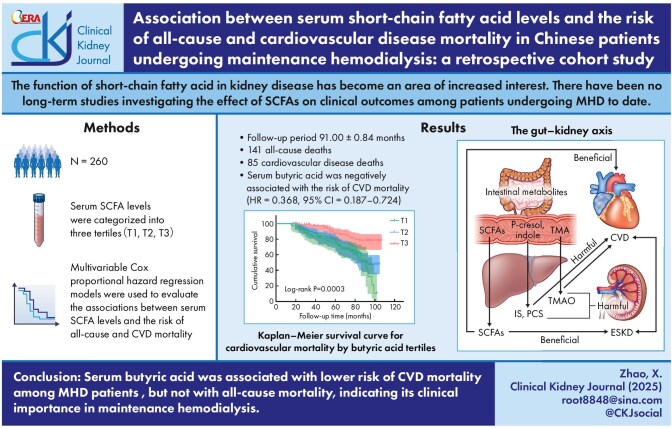

## INTRODUCTION

End-stage kidney disease (ESKD) is a major chronic condition that endangers human health and consumes a large amount of health resources. The International Society of Nephrology's 2019 Global Kidney Health Atlas cross-sectional survey of 160 participating countries provides incidence information on treated ESKD in 79 countries, where the average number of new ESKD diagnoses worldwide was 144 individuals per million general population [1]. China's blood purification registration system showed that 916 000 patients were undergoing hemodialysis by the end of 2023. Therefore, improving the quality of life of these patients and reducing their hospitalization and mortality risk has important clinical and social significance.

Cardiovascular disease (CVD) is the main cause of death in patients with ESKD. Epidemiological studies have revealed that over 50% of ESKD patients die from CVDs. Moreover, patients with ESKD have a 10–20 times higher risk of CVD death compared with healthy individuals of the same age group [2–4]. Patients with ESKD have a higher incidence of myocardial hypertrophy, cardiomyopathy, heart failure and coronary heart disease, leading to a higher CVD risk [5, 6]. Traditional CVD risk factors, such as diabetes, hypertension and hyperlipidemia, are only partially responsible for the increased CVD risk. A growing number of studies have shown that non-traditional risk factors, such as toxin accumulation (especially protein-bound uremic toxins), inflammation, oxidative stress and endothelial dysfunction, played a key role in CVD in patients with ESKD [7–9]. In recent years, intervention in non-traditional CVD factors in patients with ESKD has emerged as a research field. Moreover, regulating inflammation, intestinal metabolites, and protein binding uremic toxins by means of intervening in the gut–kidney axis can reduce the risk of CVD in ESKD patients [10–12]. Compared with the gut microbiota of healthy individuals, changes in the gut microbiota of patients with ESKD manifested as a decrease in the abundance of short-chain fatty acids (SCFAs)-producing bacteria, and an increase in the bacteria producing indole and p-cresol, such as Enterobacteriaceae [13, 14]. This revolutionary change in the composition of gut microbiota leads to the influx of circulating urea and other uremic toxins into the intestinal cavity, dysfunction of the intestinal epithelial barrier, and translocation of gut bacterial DNA and uremic toxins into the systemic circulation, resulting in systemic inflammation [15–17]. Gut microbiota and its metabolites interact with the host via various pathways, including the trimethylamine/trimethylamine N-oxide (TMAO), SCFA, and primary and secondary bile acid pathways. At present, a large number of studies have confirmed that TMAO is associated with an increased risk of CVD in patients with ESKD [18, 19]. However, the association between SCFAs and CVD and all-cause mortality in maintenance hemodialysis (MHD) patients is not explicit. SCFAs, also known as volatile fatty acids, are organic fatty acids with a carbon atom number of 1–6 in the carbon chain. SCFAs are naturally produced in small amounts in the liver, typically as the main bacterial metabolites generated by dietary fiber fermentation and resistant starch through specific gut microbiota in the colon [20, 21]. Although their main site of action is the intestine itself, they can be absorbed through the intestinal wall and portal vein and reach systemic circulation and distribute to other body organs and tissues for metabolism (although at a lower level) [22, 23].

A prior study showed that a higher level of plasma valerate, a SCFA generated by the gut microbiota, was independently associated with pre-existing CVD in patients with chronic kidney disease (CKD) [24]. A single-center non-randomized pilot study indicated that sodium propionate supplementation in MHD patients reduced pro-inflammatory parameters and oxidative stress and improved insulin resistance and iron metabolism. Furthermore, sodium propionate effectively lowered the level of indoxyl and p-cresol sulfate which were considered to be important gut-derived uremic toxins [25]. Several potential biological mechanisms have been proposed to explain the protective function of SCFA in CVD, including anti-inflammation, anti-atherosclerosis, oxidative stress reduction, and myocardial hypertrophy and fibrosis inhibition [26–29]. The *in vitro* and *in vivo* animal studies have shown that SCFAs may have a significant role in CVD and may decrease mortality rate observed in MHD patients.

There have been no long-term studies investigating the effect of SCFAs on clinical outcomes among Chinese patients undergoing MHD to date. Therefore, the present retrospective cohort study was conducted to explore the association between serum SCFA levels and all-cause and CVD mortality at a large hemodialysis center.

## MATERIALS AND METHODS

### Study design and population

**Figure 1:**
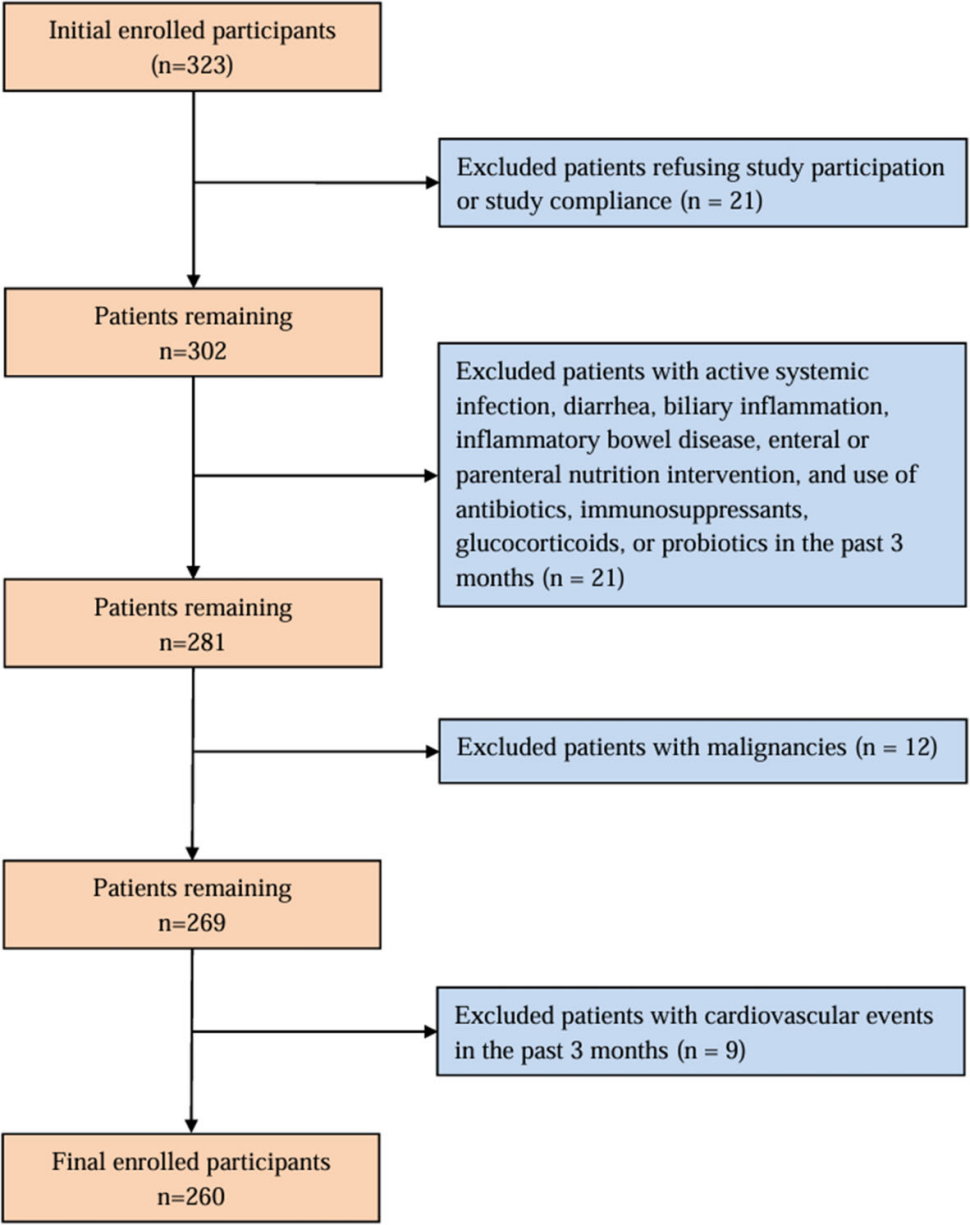
Participants inclusion chart.

The present retrospective cohort study enrolled 323 newly admitted hemodialysis patients between January 2015 and December 2017 at Central Hospital of Dalian University of Technology. The inclusion criteria were as follows: (i) patients aged 18 years or older; and (ii) individuals who received stable hemodialysis three times weekly and who had complete medical records. The exclusion criteria were as follows: (i) those refusing study participation or study compliance (*n* = 21); (ii) patients with active systemic infection, diarrhea, biliary inflammation, inflammatory bowel disease, enteral or parenteral nutrition intervention, and use of antibiotics, immunosuppressants, glucocorticoids or probiotics in the past three months (*n* = 21); (iii) patients with malignancies (*n* = 12); and (iv) those with cardiovascular events in the past 3 months (*n* = 9). Finally, 260 MHD patients were enrolled in the study (Fig. 1). All participants received maintenance dialysis for 4 h three times per week. The blood flow rate was 200–300 mL/min, and the dialysate flow rate was 500 mL/min. All participants maintained similar dietary patterns over the past 3 months and adhered to their previous exercise routine. All study protocols conformed to the principles of the Declaration of Helsinki and were approved by the Institutional Medical Ethics Committee of Central Hospital of Dalian University of Technology (protocol number: YN2022-039-17, date of ethical approval: 26 May 2022). All study patients provided written informed consent to participate.

### Data collection

Baseline demographic and clinical information [age, gender, height, weight, body mass index (BMI), primary cause of ESKD, smoking status and drinking status, and comorbidities, such as diabetes (DM), hypertension and coronary artery disease (CAD)] was collected. A physical examination was performed after dialysis to measure the patients’ weight. Body height was measured by trained personnel according to a standard protocol. The formula for calculating BMI was as follows: BMI = weight in kilograms divided by the square of the height in meters (kg/m^2^). Smoking was defined as “yes” if the participant smoked more than one cigarette per day over 6 months. Drinking was described as “yes” if the participant consumed alcohol once or more per week for over 6 months. Blood was collected before dialysis during the midweek dialysis day 3 months after its commencement. Blood was sampled immediately using a 5-mL separating gel accelerator tube and a 5-mL ethylenediamine tetraacetic acid anticoagulation tube. Blood samples were processed (centrifugation at 3500 r.p.m. for 5 min) within 30 min of sampling to obtain plasma, while serum was allowed to clot for 30 min at room temperature prior to centrifugation (3500 r.p.m., 5 min) and stored at −80°C until further use in the assays. Blood urea nitrogen, albumin, creatinine, lipid and ferritin levels were measured at an on-site biochemistry laboratory using standard autoanalyzer techniques. Intact parathyroid hormone concentration was measured using electrochemiluminescence immunoassay. Hemoglobin level was determined using sodium dodecyl lauryl sulfate. The SCFA levels in serum samples were quantified using gas chromatography–mass spectrometry with a TRACE™ 1310‐ISQ LT GC system (Thermo Fisher Scientific, Waltham, MA, USA) and conducted by BioNovoGene Co., Ltd. The SCFAs included acetic, propionic, butyric, isobutyric, valeric, isovaleric and caproic acids.

### Outcome evaluation

Patients were followed up until December 2023. The primary outcome was all-cause and CVD mortality during the follow-up period. CVD-related deaths included those resulting from sudden cardiac death, heart failure, myocardial infarction and serious arrhythmias. All deaths and events were recorded using Therapy Support Suite 2.0 (Baden Humboldt, German), B-Soft Enterprise Application Portal 5.5 (Hangzhou, China) and Chinese National Renal Data System 2017 (Beijing, China). For each patient, the time to event was calculated as the time from the date of entry into the study to the date of death, the time of kidney transplantation, the date of quitting the study or the end of the study, whichever came first.

### Statistical analysis

Serum SCFA levels were categorized into three tertiles, and the lowest tertile served as the reference group. The normality of all continuous variables was evaluated using the Shapiro–Wilk statistic. Continuous variable results were expressed as mean ± standard deviation, and intergroup comparisons were analyzed by one-way analysis of variance for normally distributed data or Kruskal–Wallis H test for non-normally distributed data. Categorical variables were expressed as counts with percentages. Differences between the three groups were examined using chi square test. Survival curves were calculated using the Kaplan–Meier method, and differences between the curves were analyzed using the log-rank test. The Schoenfeld residual test was utilized to verify the assumption of proportional hazards in the Cox analysis and no violations were found (all *P* > .05). Multivariable Cox proportional hazard regression models were used to calculate the hazards ratios (HRs) and the corresponding 95% confidence intervals (CIs). The models were without any crude adjustment. Model 2 was adjusted for age and gender. Model 3 was adjusted for age, gender, BMI, hypertension, diabetes, smoking and drinking status, and levels of hemoglobin, albumin, creatinine, cholesterol, high-density lipoprotein cholesterol and low-density lipoprotein cholesterol. Subgroup analyses based on age, gender, diabetes, CAD and BMI were also performed. The dose–response relationship between butyric acid and cardiovascular mortality was assessed with a restricted cubic spline (RCS) regression model. Statistical significance was set at *P* < .05 and was based on a two-sided test. All statistical analyses were performed using GraphPad Prism 9 (GraphPad Software, San Diego, CA, USA) and SPSS 27 (IBM, Armonk, NY, USA).

## RESULTS

The follow-up duration was (91.00 ± 0.84) months. There were 141 deaths during the follow-up period, of which 85 were attributed to CVDs. The baseline patient characteristics are shown in Table 1. The number of male patients (61.54%) was higher than the number of female (38.46%) patients. The median age of the participants was 59 years (interquartile range 47–69 years). Among all patients, 89.62% had hypertension, 50.76% had diabetes and 15.77% had CAD. The prevalence rates of diabetic nephropathy, glomerulonephritis, hypertensive benign renal arteriosclerosis and other disorders were 45.38%, 27.69%, 13.85% and 13.08%, respectively.

**Table 1:** Baseline characteristics of the study patients according to serum butyric acid levels.

		Butyric acid range (μg/mL)			
	Study population	T1 (<0.05116)	T2 (0.05116, 0.08393)	T3 (>0.08393)	
Characteristics	(*n* = 260)	(*n* = 86)	(*n* = 87)	(*n* = 87)	*P*
Age (years)	57.37 ± 15.03	59.76 ± 15.85	57.56 ± 14.33	54.83 ± 14.64	.096
Gender (male %)	61.54	61.63	59.77	63.22	.896
Height (m)	1.69 ± 0.07	1.70 ± 0.07	1.69 ± 0.07	1.69 ± 0.07	.570
Weight (kg)	70.62 ± 13.40	70.24 ± 12.42	72.62 ± 14.06	69.99 ± 13.55	.192
BMI (kg/m^2^)	24.67 ± 4.01	24.31 ± 3.46	25.52 ± 4.55	24.16 ± 3.83	.050
Primary disease (%)					
Diabetic nephropathy	45.38	47.67	49.43	39.08	.341
Glomerulonephritis	27.69	25.58	21.84	35.63	.110
Hypertensive benign renal arteriosclerosis	13.85	13.95	11.49	16.09	.680
Others	13.08	12.79	17.24	9.20	.288
Diabetes mellitus (%)	50.76	58.14	55.17	39.08	.026
Hypertension (%)	89.62	91.86	90.80	86.21	.431
Coronary heart disease (%)	15.77	20.93	16.09	10.34	.161
Smoking (%)	33.85	24.42	35.63	41.38	.057
Drinking (%)	15.77	5.81	18.39	22.99	.006
Acetic acid (μg/mL)	4.4606 ± 2.6847	4.2195 ± 1.9150	4.1293 ± 2.4665	5.0303 ± 3.3913	.051
Propionic acid (μg/mL)	0.1872 ± 0.13 929	0.1645 ± 0.1106	0.1737 ± 0.1373	0.2230 ± 0.1597	.011
Isobutyric acid (μg/mL)	0.0632 ± 0.0765	0.0292 ± 0.0296	0.0789 ± 0.0808	0.0812 ± 0.0922	.000
Valeric acid (μg/mL)	0.0104 ± 0.0095	0.0102 ± 0.0082	0.0106 ± 0.0106	0.0105 ± 0.0097	.343
Isovaleric acid (μg/mL)	0.0160 ± 0.0103	0.0172 ± 0.0106	0.0149 ± 0.0090	0.0159 ± 0.0112	.952
Hexanoic acid (μg/mL)	0.0102 ± 0.0092	0.0095 ± 0.0075	0.0104 ± 0.1039	0.0108 ± 0.0096	.621
Hemoglobin (g/L)	85.20 ± 20.27	89.04 ± 20.42	84.95 ± 19.62	81.66 ± 20.30	.055
Albumin (g/L)	34.92 ± 5.50	34.95 ± 5.54	34.90 ± 6.04	34.91 ± 4.93	.998
Creatine (μmol/L)	779.58 ± 335.34	778.93 ± 332.01	751.95 ± 345.78	807.83 ± 329.49	.548
Urea nitrogen (mmol/L)	28.48 ± 12.66	27.60 ± 11.36	27.93 ± 12.65	29.90 ± 13.86	.438
Triglyceride (mmol/L)	1.53 ± 1.30	1.41 ± 0.69	1.65 ± 1.81	1.54 ± 1.16	.495
Cholesterol (mmol/L)	4.41 ± 1.31	4.37 ± 1.35	4.58 ± 1.36	4.28 ± 1.22	.321
HDL-C (mmol/L)	0.94 ± 0.36	0.94 ± 0.27	0.92 ± 0.28	0.95 ± 0.48	.872
LDL-C (mmol/L)	2.50 ± 1.00	2.59 ± 1.06	2.59 ± 0.97	2.32 ± 0.98	.131
iPTH (pg/mL)	388.51 ± 326.00	475.13 ± 419.68	343.97 ± 275.95	347.42 ± 242.12	.010
Ferritin (ng/mL)	264.45 ± 296.92	233.47 ± 264.96	260.96 ± 287.17	264.45 ± 296.92	.352

Data are presented as means and standard deviations for continuous variables, and as frequencies and percentages for categorical variables.

BMI, body mass index; iPTH, intact parathyroid hormone; HDL-C, high-density lipoprotein cholesterol; LDL-C, low-density lipoprotein cholesterol.

**Figure 2:**
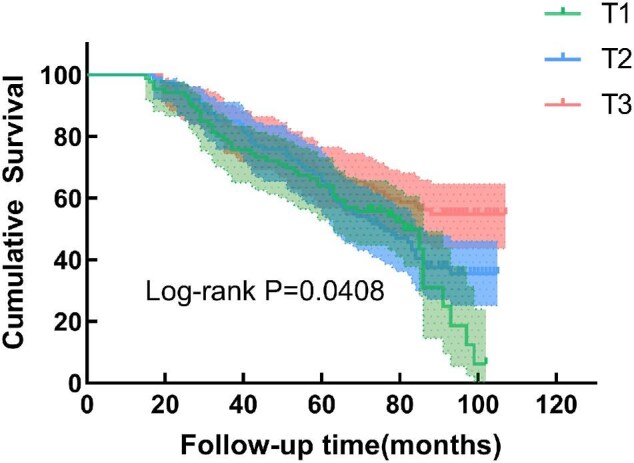
Kaplan–Meier survival curve for all-cause mortality by butyric acid tertiles.

**Figure 3:**
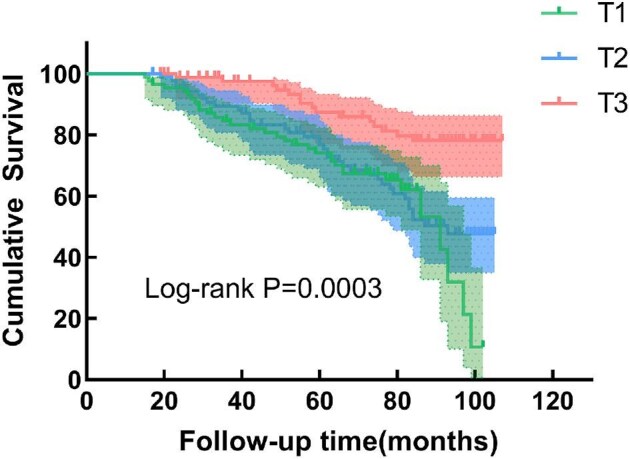
Kaplan–Meier survival curve for CVD mortality by butyric acid tertiles.

The associations between serum SCFA levels and the risk of all-cause and CVD mortality are shown in Tables 2 and 3. After adjusting for potential confounders, the level of serum butyric acid was negatively associated with the risk of CVD mortality. The highest levels of butyric acid showed a decrease in CVD mortality rate (HR 0.368, 95% CI 0.187–0.724), where a linear trend was evident (*P* < .05). Kaplan–Meier survival curves (Fig. 2) showed that the risk of CVD mortality in the highest tertile of serum butyric acid was significantly lower than that in the lowest tertile (log-rank, *P* < .05) while the same outcome was not observed in the risk of all-cause mortality (Fig. 3). However, none of the SCFAs was significantly associated with the risk of all-cause mortality after adjusting for potential confounders.

**Table 2:** HRs (95% CIs) for the association between serum SCFA levels and all-cause mortality.

Categories	Model 1^a^ HR (95% CI)	Model 2^b^ HR (95% CI)	Model 3^c^ HR (95% CI)
Acetic acid			
T1 (<3.15752)	1.00 (Ref)	1.00 (Ref)	1.00 (Ref)
T2 (3.15752, 4.84473)	1.113 (0.721–1.719)	1.098 (0.711–1.696)	1.074 (0.693–1.665)
T3 (>4.84473)	1.910 (1.273–2.867)	1.365 (0.905–2.061)	1.436 (0.935–2.206)
*P* for trend	0.002^*^	0.138	0.099
Propionic acid			
T1 (<0.11529)	1.00 (Ref)	1.00 (Ref)	1.00 (Ref)
T2 (0.11529, 0.21738)	1.064 (0.704–1.608)	0.962 (0.635–1.458)	0.950 (0.615–1.469)
T3 (>0.21738)	1.200 (0.805–1.788)	1.166 (0.781–1.741)	1.094 (0.720–1.663)
*P* for trend	0.370	0.454	0.673
Butyric acid			
T1 (<0.05116)	1.00 (Ref)	1.00 (Ref)	1.00 (Ref)
T2 (0.05116, 0.08393)	0.938 (0.634–1.390)	1.125 (0.757–1.671)	1.037 (0.686–1.570)
T3 (>0.08393)	0.603 (0.392–0.927)	0.793 (0.513–1.225)	0.739 (0.462–1.183)
*P* for trend	0.021^*^	0.296	0.208
Isobutyric acid			
T1 (<0.02230)	1.00 (Ref)	1.00 (Ref)	1.00 (Ref)
T2 (0.02230, 0.04803)	1.237 (0.833–1.838)	1.047 (0.703–1.560)	1.005 (0.664–1.520)
T3 (>0.04803)	0.691 (0.450–1.061)	0.907 (0.585–1.406)	0.856 (0.546–1.343)
*P* for trend	0.091	0.662	0.499
Valeric acid			
T1 (<0.00560)	1.00 (Ref)	1.00 (Ref)	1.00 (Ref)
T2 (0.00560, 0.00988)	0.863 (0.574–1.299)	0.922 (0.613–1.389)	0.886 (0.579–1.355)
T3 (>0.00988)	1.024 (0.688–1.524)	1.083 (0.726–1.616)	1.062 (0.706–1.595)
*P* for trend	0.907	0.696	0.774
Isovaleric acid			
T1 (<0.01030)	1.00 (Ref)	1.00 (Ref)	1.00 (Ref)
T2 (0.01030, 0.01638)	1.457 (0.967–2.194)	1.074 (0.709–1.627)	1.030 (0.670–1.582)
T3 (>0.01638)	1.356 (0.892–2.061)	1.034 (0.678–1.578)	0.971 (0.624–1.511)
*P* for trend	0.154	0.877	0.895
Caproic acid			
T1 (<0.00598)	1.00 (Ref)	1.00 (Ref)	1.00 (Ref)
T2 (0.00598, 0.01053)	0.788 (0.527–1.178)	0.741 (0.494–1.112)	0.788 (0.513–1.211)
T3 (>0.01053)	0.834 (0.559–1.244)	0.753 (0.504–1.126)	0.739 (0.486–1.123)
*P* for trend	0.374	0.167	0.156

a Model 1: crude model.

b Model 2: adjusted for age, gender.

c Model 3: adjusted for age, gender, BMI, hemoglobin, albumin, creatinine, cholesterol, high-density lipoprotein cholesterol, low-density lipoprotein cholesterol, hypertension, diabetes, smoking and drinking status.

Ref, reference; T, tertile.

HR, hazard ratio; CI, confidence interval. ^*^P<.05

**Table 3:** HRs (95% CIs) for the association between serum SCFA levels and CVD mortality.

Categories	Model 1^a^ HR (95% CI)	Model 2^b^ HR (95% CI)	Model 3^c^ HR (95% CI)
Acetic acid			
T1 (<3.15752)	1.00 (Ref)	1.00 (Ref)	1.00 (Ref)
T2 (3.15752, 4.84473)	1.177 (0.666–2.080)	1.136 (0.643–2.007)	1.130 (0.636–2.005)
T3 (>4.84473)	2.193 (1.293–3.720)	1.587 (0.927–2.718)	1.859 (1.066–3.243)
*P* for trend	0.004^*^	0.092	0.029^*^
Propionic acid			
T1 (<0.11529)	1.00 (Ref)	1.00 (Ref)	1.00 (Ref)
T2 (0.11529, 0.21738)	1.044 (0.607–1.795)	0.970 (0.561–1.675)	0.891 (0.504–1.575)
T3 (>0.21738)	1.311 (0.788–2.179)	1.309 (0.784–2.185)	1.324 (0.774–2.265)
*P* for trend	0.297	0.304	0.306
Butyric acid			
T1 (<0.05116)	1.00 (Ref)	1.00 (Ref)	1.00 (Ref)
T2 (0.05116, 0.08393)	0.893 (0.555–1.439)	1.040 (0.643–1.683)	0.975 (0.589–1.615)
T3 (>0.08393)	0.316 (0.170–0.589)	0.412 (0.220–0.771)	0.368 (0.187–0.724)
*P* for trend	0.000^*^	0.006^*^	0.004^*^
Isobutyric acid			
T1 (<0.02230)	1.00 (Ref)	1.00 (Ref)	1.00 (Ref)
T2 (0.02230, 0.04803)	1.030 (0.616–1.721)	0.859 (0.512–1.442)	0.790 (0.461–1.351)
T3 (>0.04803)	0.651 (0.380–1.115)	0.848 (0.489–1.470)	0.743 (0.421–1.310)
*P* for trend	0.118	0.557	0.304
Valeric acid			
T1 (<0.00560)	1.00 (Ref)	1.00 (Ref)	1.00 (Ref)
T2 (0.00560, 0.00988)	0.910 (0.541–1.532)	0.973 (0.577–1.641)	1.021 (0.595–1.752)
T3 (>0.00988)	1.015 (0.605–1.703)	1.059 (0.629–1.782)	1.071 (0.631–1.820)
*P* for trend	0.956	0.829	0.799
Isovaleric acid			
T1 (<0.01030)	1.00 (Ref)	1.00 (Ref)	1.00 (Ref)
T2 (0.01030, 0.01638)	0.958 (0.557–1.646)	0.710 (0.410–1.228)	0.629 (0.357–1.108)
T3 (>0.01638)	1.311 (0.791–2.172)	0.995 (0.597–1.659)	0.845 (0.493–1.446)
*P* for trend	0.294	0.986	0.538
Caproic acid			
T1 (<0.00598)	1.00 (Ref)	1.00 (Ref)	1.00 (Ref)
T2 (0.00598, 0.01053)	0.835 (0.492–1.419)	0.758 (0.444–1.294)	0.792 (0.449–1.398)
T3 (>0.01053)	1.012 (0.606–1.689)	0.908 (0.543–1.519)	0.863 (0.503–1.482)
*P* for trend	0.964	0.712	0.594

a Model 1: crude model.

b Model 2: adjusted for age, gender.

c Model 3: adjusted for age, gender, BMI, hemoglobin, albumin, creatinine, cholesterol, high-density lipoprotein cholesterol, low-density lipoprotein cholesterol, hypertension, diabetes, smoking and drinking status.

Ref, reference; T, tertile.

HR, hazard ratio; CI, confidence interval. ^*^P<.05

**Figure 4:**
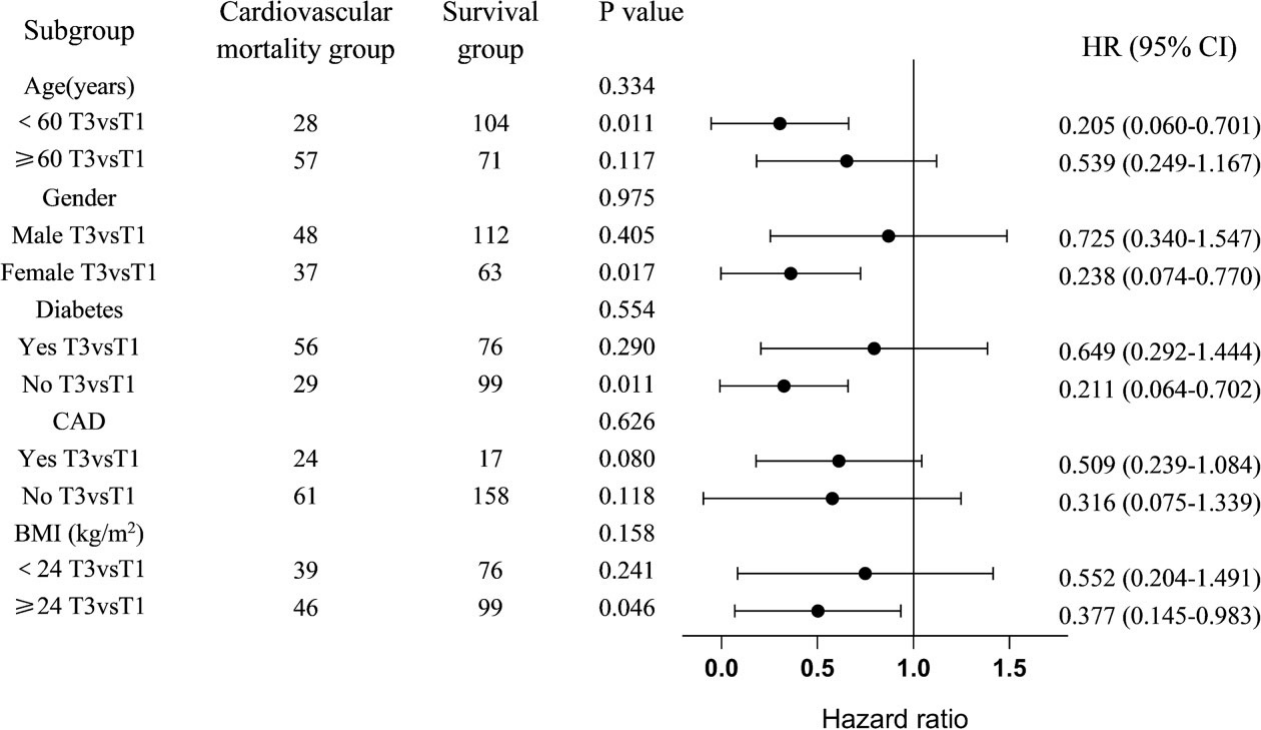
Stratified analyses for the adjusted HR and 95% CI for the association between butyric acid and CVD mortality.

Subgroup analysis indicated that the association between serum butyric acid and CVD mortality in participants divided into groups based on age, gender, diabetes status, CAD status and BMI showed no significant differences (*P* > .05; Fig. 4). These analyses demonstrated that the association between butyric acid and CVD mortality is reliable and stable. Although no statistically significant interaction was observed in subgroup analysis, the levels of serum butyric acid were negatively associated with the risk of CVD mortality in the young and middle-aged patients (aged below 60 years), female individuals, non-diabetic participants and those with BMI exceeding 24 kg/m^2^. The highest levels of butyric acid in these patients showed a decrease in CVD mortality rate (HR 0.205, 95% CI 0.060–0.701; HR 0.238, 95% CI 0.074–0.770; HR 0.211, 95% CI 0.064–0.702; HR 0.377, 95% CI 0.145–0.983, respectively) with an evident linear trend (*P* < .05).

**Figure 5:**
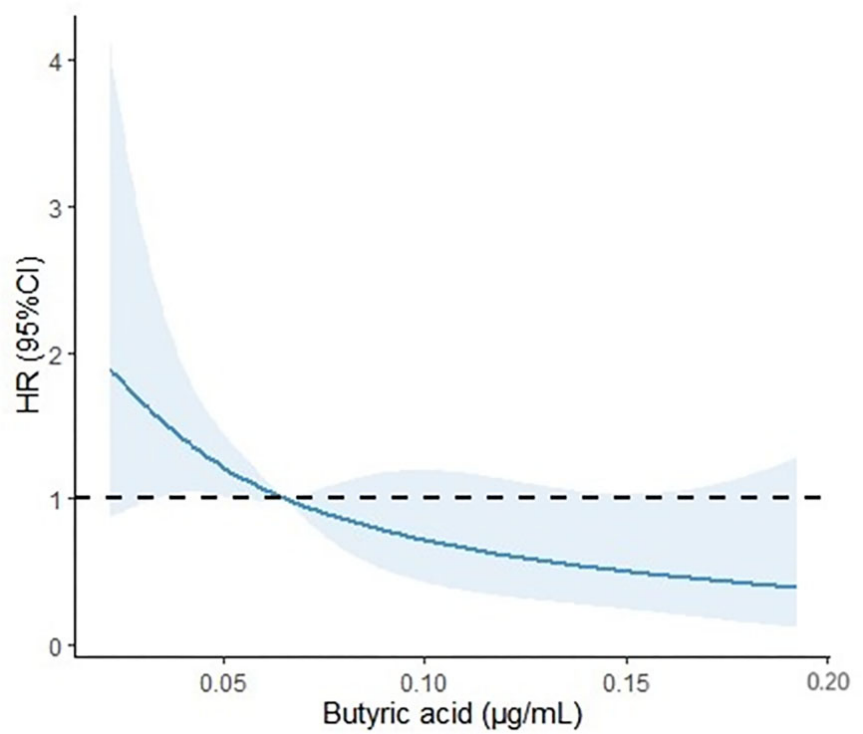
The dose–response curve of the relationship between butyric acid and CVD mortality.

An RCS regression model was applied to determine whether a nonlinear relationship existed between butyric acid and CVD mortality. Butyric acid was negatively associated with CVD mortality. Thus, the likelihood of CVD mortality decreased with increasing butyric acid level (*P*_nonlinear _> .05; Fig. 5, the blue line and shaded area represent the estimated HR and 95% CI).

## DISCUSSION

The present observational study examined the associations between serum SCFA levels and the risk of all-cause and CVD mortality among MHD patients. The findings indicated that higher serum butyric acid level was independently associated with lower risk of CVD mortality, but not with all-cause mortality after adjusting for potential confounders.

An increase in phyla Actinobacteria, Firmicutes and Proteobacteria and a decrease in *Bifidobacteria* and *Lactobacilli* and SCFA levels have been reported in the course of CKD and in patients with ESKD [30, 31]. These factors serve as evidence that SCFA levels progressively decrease during the different stages of CKD and, ultimately, in dialysis patients [32]. This change in the composition of gut microbiota leads to the influx of uremic toxins into the intestinal cavity and dysfunction of the intestinal epithelial barrier, resulting in systemic inflammation. Uremic toxins and systemic inflammation can cause damage to vascular endothelial cells [15–17].

Only a few studies have explored the associations between SCFA and the incidence of CVD in patients with CKD. In a sub-cohort of 214 patients with CKD, including 81 patients with CAD and CVD, it was demonstrated that a higher level of plasma valerate was independently associated with pre-existing CVD in patients with CKD [24]. However, the present study did not reveal any correlation between valeric acid and CVD mortality. The inconsistency between these results may be due to the differences in observational outcome. Valeric acid has not been studied as extensively as other SCFAs, and details of its physiological effects, especially the impact on CVD, are sparse.

A pilot study recruited 20 stable MHD patients suffering from vascular and cardiac complications. After 12 weeks of treatment with food additive sodium propionate, a significant decline including TGF-β, malondialdehyde, indoxyl sulfate and p-cresyl sulfate levels, was demonstrated [25]. These parameters are important pathogenetic factors of CVD in ESKD patients [33, 34]. Therefore, sodium propionate supplementation was assumed to be beneficial for CVD treatment in patients with ESKD. However,

the benefits of propionic acid on CVD were not observed in the present study, which may be due to the differences in sample size and study design.

A cross-sectional study revealed that SCFA levels in the feces of patients with atrial fibrillation were decreased compared with those in healthy individuals [35]. A longitudinal prospective study recruited 18 newly diagnosed heart failure patients, and the results showed that remission of heart failure may have association with the reversal of gut microbiota dysbiosis and an increase in SCFA production, especially butyrate [36]. Although the present study participants were different from the patients in the aforementioned studies, these results may shed light on the main findings in the current investigation.

There is increasing evidence showing that SCFAs, especially butyrate acid, can exert cardiovascular protective effects via the gut–kidney–heart axis in animal studies. First, GPR41 stimulation by butyrate regulates blood pressure homeostasis [37]. Second, sodium butyrate significantly inhibits histone deacetylase (HDAC) and activates the phosphorylation of MKK3/P38/PRAK inhibiting myocardial cell hypertrophy [38]. Sodium butyrate can effectively reduce the activity of class I HDACs and limit myocardial remodeling and fibrosis [39]. Butyrate can rapidly correct mitochondrial adenosine 5′ triphosphate synthesis, regulating myocardial energy metabolism and improving the contractile function [40]. Third, butyrate slows the progression of atherosclerosis, which is linked to the reduction in CD36 levels in macrophages and endothelial cells, decline in pro-inflammatory cytokine levels, and lower nuclear factor-κB activation level [41]. Finally, butyrate enhances epithelial cell proliferation and improves tight junctions of the mucus layer, maintaining intestinal barrier integrity [42]. Butyrate protects intestinal barrier function by inhibiting NLRP3 (cytosolic protein) inflammasome [43, 44]. It also protects epithelial cells from lumen toxins via the expression of the *MUC2* gene [45]. The activation of butyrate-mediated GPR43 attenuates renal inflammation, reduces uremic toxin levels, such as those of p-cresyl sulfate and indoxyl-sulfate, and delays CKD progression [46]. Therefore, butyric acid can be regarded as a protective factor against cardiovascular deaths in patients with ESKD.

Simultaneously, the present subgroup analysis revealed that elevated butyric acid levels exhibit a protective effect against cardiovascular mortality in young and middle-aged patients (aged below 60 years), female individuals, non-diabetic participants and those with BMI exceeding 24 kg/m². The diversity of intestinal flora and butyric acid production decrease in the elderly patients [47–50], while other cardiovascular risk factors, such as vascular calcification and atherosclerosis, are more serious than those in young and middle-aged patients [51–53]. This may lead to the weakening of the protective effect of butyric acid on cardiovascular death.

Estrogen is a main regulatory factor of gut microbiota, modulating the composition of gut microbiome by promoting the growth of bacteria that produce butyric acid [54–56]. It exerts its effects by inhibiting inflammatory signaling pathways [57, 58], enhancing intestinal barrier function, and improving energy metabolism, which may have enhanced the cardiovascular protective effect of butyric acid in female patients in the present study. A recent meta-analysis showed that CVD mortality in DM dialysis patients is 2.11-fold greater compared with that in non-DM dialysis patients [59]. SCFA-producing bacteria levels in diabetes patients are significantly reduced, which may damage the activation of SCFA receptors and affect the anti-inflammatory response of the intestine. Non-diabetes patients may retain a more complete role of intestinal metabolites in regulating cardiovascular function [60, 61]. It has been confirmed that patients with diabetic nephropathy who are on hemodialysis tend to have worse left ventricular diastolic function and more frequent heart valve problems [62]. In T2DM, advanced glycation end products accumulation triggers oxidative stress and inflammation and promotes atherosclerosis [63, 64]. Due to the diversity of cardiovascular risk factors in patients with diabetes, butyric acid may not be critical to their cardiovascular protection and may not play a reversing role. Some studies have reported a positive correlation between fecal SCFA concentrations and obesity [63, 65, 66]. During inflammatory conditions or malnutrition, body protein stores are diverted to defend against inflammation and to repair injury. The increased body mass in overweight dialysis patients offers protection against or resources for responding to inflammation, infection and CVD [67]. The higher BMI, through mechanisms beyond better nutrition, may offset part of the uremic toxin effect in uremic patients [68]. Therefore, patients with a high BMI may have a protective effect on cardiovascular mortality risk due to its impact on nutritional reserves. Butyric acid may amplify this effect.

The present study has several strengths. First, it takes the lead in investigating the role of serum SCFA levels when predicting the risk of all-cause and CVD mortality in MHD patients in China. Second, this retrospective study was conducted in a well-characterized cohort of MHD patients from which detailed demographic and clinical information on comorbidities and laboratory indicators was collected. This allowed to explore the associations between serum SCFA levels and the risk of all-cause and CVD mortality with greater accuracy. Finally, an RCS regression model was used to estimate the detailed dose–response relationship between serum butyric acid and CVD mortality, thus providing practical suggestions to reduce the risk of CVD. The present study raised the awareness among MHD patients about how SCFA may be beneficial for CVD.

However, there were also several limitations in the study. First, serum SCFA levels were measured at a single time point, which may not reflect substantial intra-individual variability over time. These findings can provide some clues for future studies. Second, due to the limited data, the present study may have been influenced by some unmeasured confounders, such as residual renal function and N-terminal pro-B-type natriuretic peptide. Although blood lipid levels, albumin level, BMI and SCFA levels were incorporated as indicators of patients’ metabolic status, other metabolic biomarkers, such as waist-to-hip ratio, body fat percentage, visceral fat content, adipokines and myokines, were omitted. Third, the enrolled patients were all from a single center. Thus, the results may not be extrapolated to the overall MHD population. In addition, the relatively small sample size and short follow-up duration might have caused some analyses to be insufficiently informative. Therefore, future studies that record SCFA levels in hemodialysis patients at multiple time points and centers may help to verify the potential utility of clinical interventions that target SCFA.

## CONCLUSIONS

In conclusion, the present study demonstrated that serum butyric acid was associated with lower risk of CVD mortality among MHD patients, but not with all-cause mortality, indicating its clinical importance in MHD. The function of gut microbiota–derived SCFAs in kidney disease has become an area of increased interest in recent years and further prospective large-scale studies are needed to confirm the study findings.

## Data Availability

The datasets used and/or analyzed during the present study are available from the corresponding author upon reasonable request.
